# Multi-Site Photoplethysmography Technology for Blood Pressure Assessment: Challenges and Recommendations

**DOI:** 10.3390/jcm8111827

**Published:** 2019-11-01

**Authors:** Gabriel Chan, Rachel Cooper, Manish Hosanee, Kaylie Welykholowa, Panayiotis A. Kyriacou, Dingchang Zheng, John Allen, Derek Abbott, Nigel H. Lovell, Richard Fletcher, Mohamed Elgendi

**Affiliations:** 1 Faculty of Medicine, University of British Columbia, Vancouver, BC V6T 1Z3, Canada; gabriel.chan@alumni.ubc.ca (G.C.); rachelcooper@alumni.ubc.ca (R.C.); manish.hosanee@alumni.ubc.ca (M.H.); kayliewelykholowa@gmail.com (K.W.); 2 School of Mathematics, Computer Science and Engineering, University of London, London EC1V 0HB, UK; p.kyriacou@city.ac.uk; 3 Research Center of Intelligent Healthcare, Faculty of Health and Life Science, Coventry University, Coventry CV1 5FB, UK; dingchang.zheng@coventry.ac.uk; 4 Microvascular Diagnostics, Northern Medical Physics and Clinical Engineering, Freeman Hospital, Newcastle upon Tyne NE7 7DN, UK; john.allen@ncl.ac.uk; 5 School of Electrical and Electronic Engineering, The University of Adelaide, Adelaide, SA 5005, Australia; derek.abbott@adelaide.edu.au; 6 Centre for Biomedical Engineering, The University of Adelaide, Adelaide, SA 5005, Australia; 7 Graduate School of Biomedical Engineering, UNSW Sydney, Sydney, NSW 2052, Australia; n.lovell@unsw.edu.au; 8 D-Lab, Massachusetts Institute of Technology, Cambridge, MA 02139, USA; fletcher@media.mit.edu; 9 Department of Psychiatry, University of Massachusetts Medical School, Worcester, MA 01655, USA; 10 School of Electrical and Computer Engineering, University of British Columbia, Vancouver, BC V6T 1Z4, Canada; 11 BC Children’s & Women’s Hospital, Vancouver, BC V6H 3N1, Canada

**Keywords:** photoplethysmography, digital health, global health, intensive care unit, anesthesia, wearable devices, pulse arrival time, pulse transit time, pulse wave, pulse oximeter, hypertension assessment

## Abstract

Hypertension is one of the most prevalent diseases and is often called the “silent killer” because there are usually no early symptoms. Hypertension is also associated with multiple morbidities, including chronic kidney disease and cardiovascular disease. Early detection and intervention are therefore important. The current routine method for diagnosing hypertension is done using a sphygmomanometer, which can only provide intermittent blood pressure readings and can be confounded by various factors, such as white coat hypertension, time of day, exercise, or stress. Consequently, there is an increasing need for a non-invasive, cuff-less, and continuous blood pressure monitoring device. Multi-site photoplethysmography (PPG) is a promising new technology that can measure a range of features of the pulse, including the pulse transit time of the arterial pulse wave, which can be used to continuously estimate arterial blood pressure. This is achieved by detecting the pulse wave at one body site location and measuring the time it takes for it to reach a second, distal location. The purpose of this review is to analyze the current research in multi-site PPG for blood pressure assessment and provide recommendations to guide future research. In a systematic search of the literature from January 2010 to January 2019, we found 13 papers that proposed novel methods using various two-channel PPG systems and signal processing techniques to acquire blood pressure using multi-site PPG that offered promising results. However, we also found a general lack of validation in terms of sample size and diversity of populations.

## 1. Introduction

Hypertension (HTN), defined as a blood pressure (BP) above 140/90 mmHg on two or more occasions, is one of the most prevalent diseases, affecting over 1 billion people world-wide [1]. The European Society of Cardiology and the European Society of Hypertension estimates that by 2025, the number of affected people will be 1.5 billion [1]. HTN is often called the “silent killer” because there are usually no symptoms, but it is associated with chronic kidney disease, cardiovascular disease, and death [2]. In addition, hypertension in pregnancy is associated with preterm birth, still birth, intrauterine growth restrictions, neonatal thrombocytopenia, and bronchopulmonary dysplasia [3]. Therefore, early detection and intervention are critical.

### 1.1. The Need for a Non-Invasive Continuous Blood Pressure Monitor

The current industry gold standards of blood pressure assessment are the manual sphygmomanometer (BP cuff) and invasive blood pressure (IBP) measurements [4]. The invasive arterial line is currently only used in high-risk surgical patients or critically ill patients who require instantaneous BP measurements [5]. As expected, it is not used outside of the inpatient setting because of the risks associated with arterial cannulation, such as temporary occlusion, infection, or vascular damage [5]. The cuff-based sphygmomanometer is the most frequently used method of BP measurement in hospitals, in family practice offices, and at home. While the manual auscultatory method is the gold standard, the automatic BP cuff is also often used because of its convenience. The automatic BP cuff depends on the measurement of oscillometric changes in pressure during cuff deflation [6]. However, in both BP cuff methods, BP can only be measured intermittently and can be affected by factors such as white coat HTN, masked HTN, caffeine, exercise, or stress [7]. These measurements also provide no additional information on the cardiovascular status of the patient. Intermittent measurements using a sphygmomanometer are therefore not an ideal method for monitoring the long-term management of HTN [8]. Additionally, ambulatory home BP self-monitoring has been shown to be able to detect HTN disorders earlier than office measurements [9]. There are currently 24-h ambulatory BP monitoring (24hABPM) devices that can measure BP intermittently over the span of a day that have been shown to improve accuracy and predict cardiovascular risk. While the 24hABPM is superior to the BP cuff methods in that it can track BP trends throughout the day, it is not entirely non-invasive. The patient has to carry the device with an attached cuff that is set to inflate once every 15–20 min [10], which can be distracting and uncomfortable at the least, and could even cause microvascular damage (bruising) and pain in some patients [11]. In addition, the use of this device is costly [10]. As the prevalence of HTN increases, there is a growing need for a wearable, cuff-less, and non-invasive BP monitor that is cost-effective and can continuously monitor a patient’s blood pressure throughout day-to-day activities.

### 1.2. Photoplethysmography to Estimate BP

Photoplethysmography (PPG) is a technology that illuminates perfused tissue and then measures light that is either reflected or transmitted back to a photosensor, giving a measurement of blood volume [12]. Traditionally, PPG has been used to measure oxygen saturation in the finger, widely known as the pulse oximetry technique; however, PPG has also been shown to be able to detect changes in blood volume and the pulse wave of blood flow during systole [12]. The ability of PPG to detect the arterial pulse wave is a promising method for obtaining continuous BP measurements in a small and convenient wearable device. There have been increasing numbers of studies exploring this potential over the past decade, investigating using PPG in combination with electrocardiography (ECG), ballistocardiography (BCG), phonocardiography (PCG), impedance plethysmography (IPG), and tonometry [13]. Notably, many papers use a combination of ECG and PPG to estimate BP, with ECG used to determine the start of systole and PPG used in the periphery to determine the time taken for the pulse wave to reach a certain distance. This measured time is known as the pulse arrival time (PAT), which is defined as the time it takes the pulse wave to travel from the heart to the periphery and is proportional to BP [14].

### 1.3. Vagueness of Terminology

One of the difficulties of understanding research in PPG is the interchangeability of several key terms. This inconsistency in terminology can create confusion between research questions, protocol development, and findings, so we wanted to address it early in the review. In many publications, PAT is used interchangeably with pulse transit time (PTT). These two are not the same; the key difference is when this time interval starts. The beginning of the PAT is measured from the R wave of the ECG; therefore, it includes the pre-ejection period (PEP), which is the time it takes for blood to leave the heart after the heart’s electrical impulse [15]. PTT on the other hand is defined as the time it takes for a pulse wave to travel a known distance and does not include the PEP. It is inversely proportional to pulse wave velocity (PWV), which is the speed of the pulse wave along the arterial vessel [16]. Another point of confusion is that multi-site PPG is often used to describe a system that uses two PPG probes, but it can extend up to multiple channels [17]. In this review, we will consider dual PPG (two probes) as the key type of multi-site PPG in blood pressure assessment.

### 1.4. Multi-Site PPG

**Figure 1:**
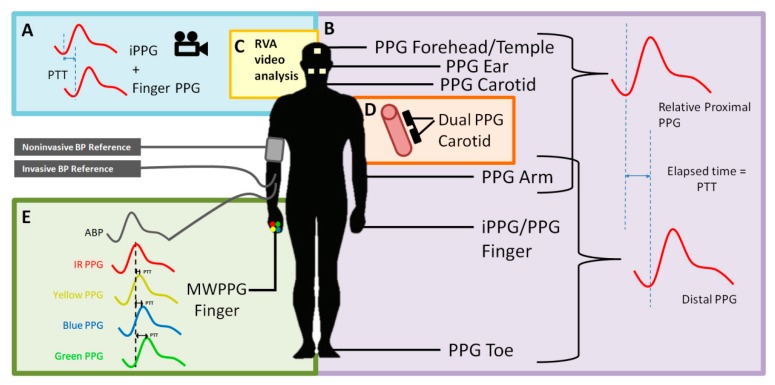
A visualization of the methods multi-site photoplethysmography (PPG) to measure the pulse transit time (PTT). Multisite PPG can make use of twos PPG probes, one proximal and one distal, to measure the time it takes for the pulse wave to travel a distance. (**A**,**B**) Two-location dual PPG/iPPG systems. (**C**) iPPG system. (**D**) Single artery dual PPG system. (**E**) Multiple wavelengths system. ECG—electrocardiography, IPG—impedance plethysmography, BCG—ballistocardiography, PCG—phonocardiography, PPG—photoplethysmography, iPPG—image-based PPG, and MWPPG—multi-wavelength PPG.

**Figure 2:**
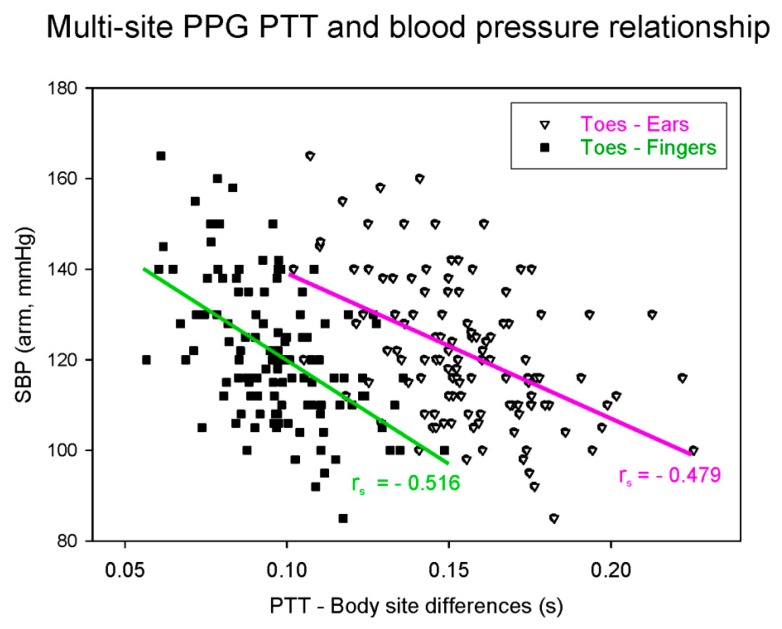
Dual probe multi-site PPG-derived pulse transit time (PTT) correlation with systolic blood pressure (SBP). The data used in this figure were adapted from Allen and Murray [19].

One of the limitations of the ECG–PPG method is that the PAT includes the PEP. This artifact reduces the accuracy of the BP estimation [15]. One of the ways to account for this is to measure the pulse wave at a proximal location rather than to measure the electrical impulse of the heart. Several studies have examined multi-site dual PPG based on just two PPG sensors, in which the ECG is replaced by a second PPG, thereby removing the confounding PEP component [16]. The resulting time interval from when the pulse wave is detected by the first PPG to when it is detected by the distal PPG is known as the pulse transit time (PTT), which is more accurately correlated to BP than the PAT [15]. The two PPG probes can be relatively proximal–distal, that is, not directly connected in the vascular tree (e.g., forehead to toe) [18], or they can be proximal–distal along the same artery (e.g., proximal brachial to distal brachial artery), as shown in Figure 1 [16]. Figure 2 shows an example of multi-site PPG being used to calculate PTT such that it is significantly correlated (*p* < 0.001) with systolic BP (SBP). Note that each data point in Figure 2 represents a subject for either PTT (ear to toe) or (finger to toe), where a total of 116 subjects were used in the analysis.

We performed a literature search for all research from January 2010 to January 2019 regarding multi-site PPG as a method for BP measurement. The purpose of this review is to gain an understanding of multi-site PPG as one of the potential technologies amongst several (Figure 1) to assess the current trends in the literature and to guide future research. We found that the amount of research in this field is limited. The majority of the studies on multi-site PPG had small sample sizes (e.g., typically 5–20 healthy subjects), with no hypertensive subjects or subjects with co-morbidities. Therefore, it appears that there is a lack of validation for this technology in clinical groups.

## 2. Methods

We conducted a thorough literature search using PubMed for articles exploring the use of multi-site PPG for BP measurement from 1/1/2010 to 1/1/2019. PubMed was used because it gave access to journal articles as well as conference proceedings. We decided to limit our search to the most recent decade, i.e., January 2010 to January 2019, because we were interested in the trend of the development of this technology in the recent decadal time frame. Note that the typical time frame is the most recent decade [20,21,22]. We used the following search terms, combined with “OR”, using PubMed’s advanced search feature: ppg blood pressure determination, ppg blood pressure estimation, ppg hypertension estimation, ppg non-invasive blood pressure, ppg cuffless blood pressure, ppg cuff-less blood pressure, photoplethysmographic blood pressure determination, photoplethysmographic blood pressure estimation, photoplethysmographic hypertension determination, photoplethysmographic hypertension estimation, non-invasive hypertension classification, non invasive blood pressure monitoring, pulse transit time blood pressure estimation, multi-ppg blood pressure, multi-site ppg blood pressure, and multi-photoplethysmographic blood pressure. Our initial searches returned a significant number of studies regarding arterial stiffness and vascular disease; we therefore added the search term “NOT arterial stiffness.” We are aware of the relatedness of vascular disease with hypertension [23]; however, including analysis of vascular disease via PPG would mean including many studies unrelated to BP. Therefore, we decided to limit the scope of our study to multi-site PPG for the analysis of BP in the hope that we could make more meaningful and BP focused recommendations. 

**Figure 3:**
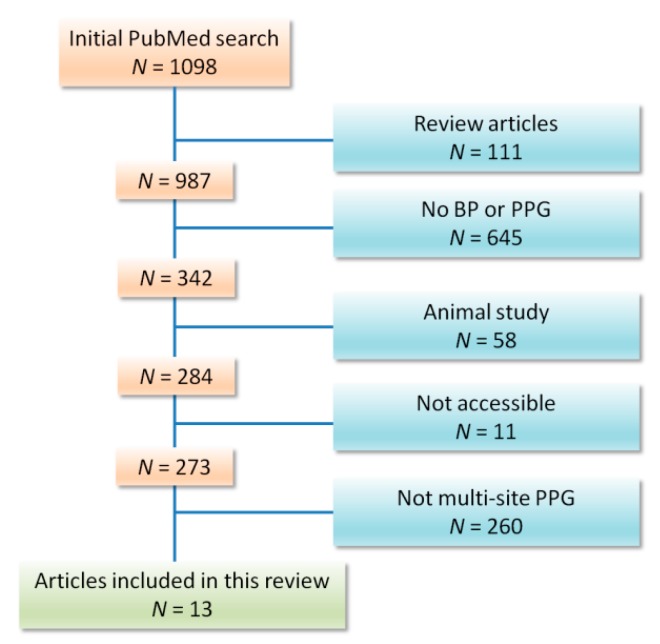
Flow chart of included studies. Thirteen studies could be included out of 1098 articles published between January 2010 and January 2019.

We included any publication that examined multi-site PPG as a method for BP measurement. Our exclusion criteria included animal studies, review articles, articles that are not accessible in the English language (see the Appendix), articles in which PPG was not used to estimate blood pressure, and articles that validated a trademarked PPG device with no discussion of the PPG technology or waveform itself. From January 2010 to January 2019, our search found 13 papers that fit the inclusion criteria (Figure 3). 

## 3. Results

**Figure 4:**
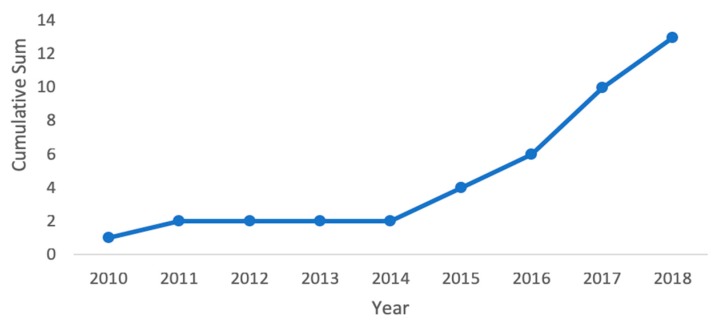
Cumulative count of multi-site PPG BP related studies from January 2010 to January 2019 showing an increase in the number of studies.

Our PubMed search found 13 publications that studied multi-site PPG technology; all but one paper looked at using two measurement sites with a two-probe system [24]. The two probes are used to measure PTT (or its inverse PWV), which is correlated with BP. From January 2010 to January 2019, multi-site PPG publications followed a growing trend (Figure 4). In our analysis, we determined the sample sizes of each paper, the inclusion of any subjects with HTN or co-morbidities, and the method used as the gold standard to collect the reference BP measurements for comparison. Of these papers, only three had a sample size of more than 30 subjects: one each in 2011 [18], 2017 [16], and 2018 [25]. In addition, most of the papers either used healthy subjects with no HTN, co-morbidities, or pregnant women, or used health statuses that were undisclosed. One paper [26] compared hypertensive (*N* = 10) and normotensive (*N* = 10) subjects, as well as patients undergoing coronary angiography (*N* = 4). Another publication collected data from patients undergoing general anesthesia (*N* = 35) [18].

### 3.1. Gold Standards

**Figure 5:**
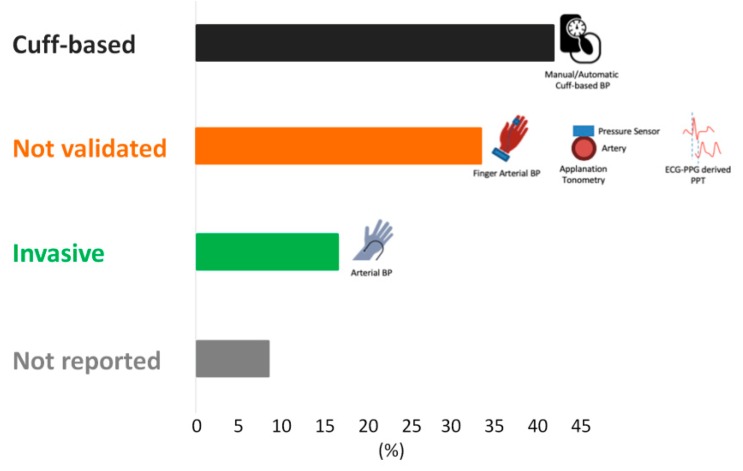
A representation of the gold standards used by each study to obtain the reference blood pressure. BP cuff—sphygmomanometer, either manual or automatic; not validated—Volume-clamp method, applanation tonometry (use of pressure sensors on the artery to detect systolic BP), or another PPG technology that has not been fully validated as the gold standard for BP measurement; invasive IBP—invasive arterial blood pressure.

Several of the papers did not use the gold standard BP measurement methods (inter-arterial line or sphygmomanometer) for their comparisons. Instead, they used less validated methods for obtaining the reference BP, such as devices that employ the volume-clamp method, other PPG methods, applanation tonometry, or no reported reference at all. The volume-clamp method, also known as finger arterial non-invasive BP, uses a PPG probe to measure the volume of blood in the fingertip and a beat-to-beat adjusting cuff that exerts pressure to maintain the volume detected by the PPG. In theory, this cuff pressure is equal to the systolic BP. Some publications have verified its validity in different applications [27], but others have shown its measurements to be different from invasive BP measurements [28]. Since the automatic sphygmomanometer is widely used in the clinical setting, for our study, we considered an automatic BP cuff as an appropriate gold standard [6]. Only eight publications used an appropriate gold standard for assessing the reference BP, where two used invasive arterial blood pressure and six used a sphygmomanometer (manual or automatic) (Figure 5)

### 3.2. Induced Changes in BP

**Figure 6:**
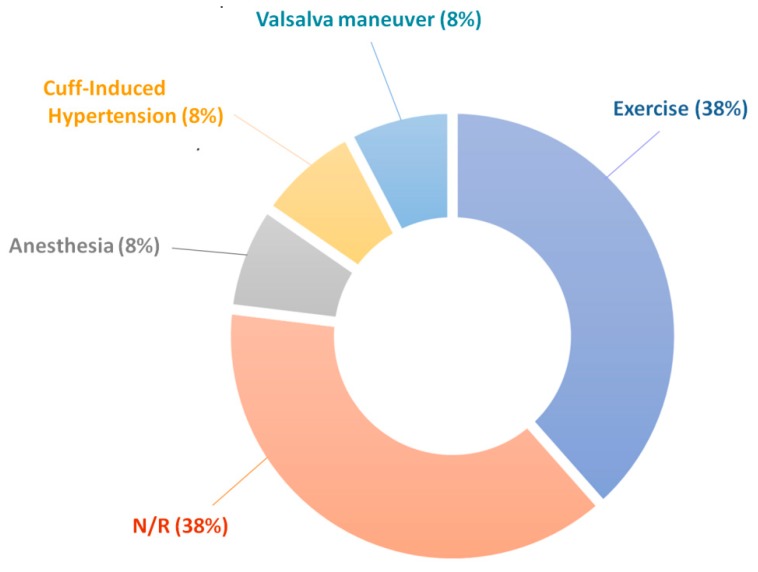
A representation of the methods used in the studies to provoke changes in blood pressure during validation of the multi-site PPG technique. Exercise = stationary calf raises, hand grip exercise, or physical activity immediately before measurement; anesthesia = general anesthesia; cuff-induced hypertension = BP cuff used to occlude the brachial artery to artificially raise the BP in the carotid; Valsalva maneuver = exhaling against a closed glottis; N/R = not reported.

Many studies included a protocol that induced changes in BP during measurement, as shown in Figure 6. In addition to physical exercise, other experimental tasks were used to drive changes in the autonomic nervous system and thus alter BP as a response through the baroreceptors and vasoconstriction. Such techniques included anesthesia, the Valsalva maneuver, a tilt table, and cold-pressor test. In other techniques, a brachial cuff was used to occlude the brachial artery, which causes a rise in blood pressure in the carotid artery where the measurements take place [29]. Using these techniques to induce changes in the blood pressure allowed the studies to show that the technology is sensitive and accurate over some range of BPs. However, the range of induced BP values is generally limited. 

### 3.3. Accuracy of BP Estimation

The accuracy of BP estimations of each analyzed publication is summarized in Table 1. There were two methods used to determine the accuracy in comparison with the reference BP: mean absolute difference and Pearson’s correlation coefficient (*r*). Three publications did not analyze their method in comparison to a reference BP. One publication expressed reliability in their measurements by showing that beat-to-beat variations of the measured PWV was less than 15% [29]. Another analyzed the correlation of PTT measurements from the wrist to different fingers (index, middle, ring, little) and found an absolute correlation value of 0.95 [30]. The third publication compared the PTT using their proposed method with PTT using the ECG–PPG method giving *r* value of 0.86 ± 0.06 [31]. Only one study found that PTT was poorly correlated with BP [32].

## 4. Discussion

Of the 13 publications, only one study, Chen et al. [32], found two-channel PTT (earlobe and finger, *N* = 20) to be poorly correlated with BP. This poor correlation is suggested to be due to the fact that the devices used had a low accuracy and sensitivity in comparison to the small changes in PTT associated with the changes in BP. In their study, the PTT varied with a massive uncertainty of ±11 ms, which was associated with a variation in the BP readings of approximately ±20 mmHg (*r* = 0.22). 

One of the studies, Beckmann et al. [30], only looked at the reproducibility of the measurements using a wrist to finger PPG (*N* = 5). This study had neither a gold standard for measuring the reference blood pressure, nor did they have any reports of accuracy of their measurements. However, they were able to show an excellent agreement within multiple measurements using different fingers (*r* = 0.95). 

Another study, Viejo et al. [24] used multi-site PPG with more than two locations, using raw video analysis of multiple locations of the face (*N* = 15). Using machine learning, they were able to create an algorithm to predict the SBP with an accuracy of *r* = 0.85, which is the most accurate SBP prediction amongst the studies included here. 

Yet another study, Chen et al. [18], used ear–toe dual PPG to measure PWV to estimate BP. The advantage of this study is that it was conducted on patients undergoing general anesthesia. In addition, this is the only study included in our analysis that examined patients of different age ranges (17–21 and 58–62 years). However, a limitation of their experiment is the small sample size. Although they had more than 30 subjects (*N* = 35), they separated their experimental validation into two separate experiments: validation of PWV (*N* = 9) and validation of BP measurement (*N* = 26). Furthermore, they had two different age groups; therefore, it would be ideal to have a larger sample size. Another limitation of the study is that there is insufficient information about the subjects. Chen et al. notes that data acquisition was done in the operating room before, during, and after anesthesia. However, how the nature of the surgeries, health of the subjects, and the use of any medication could affect the BP is unknown. 

### 4.1. Wearable PPG-Based Devices

One of the main motivators for studying multi-site PPG is its potential for incorporation into a wearable device to continuously monitor BP in an ambulatory setting. This would be designed to allow for the early detection of HTN and provide an accurate and complete profile of BP control in hypertensive patients. Based on the research found, there seem to be two major barriers to creating a wearable device. First, the device must be compact enough to be convenient to wear on a daily basis, and second, PPG measurements are usually highly sensitive to body movements, where the signal collected contains motion artifacts that can decrease the accuracy of the BP estimation.

### 4.2. Compact Design

A second study, Liu et al. [31], proposed the use of the front and back cameras on a smartphone to measure PTT. The device used in the study had one camera surrounded by a ring of light-emitting diode (LED) lights on one side and a standalone camera on the other side. The side with the LED lights was pressed against the temple while the index finger of the right hand was pressed against the second camera, in a position similar to when one is talking on a phone. The camera for the index finger was illuminated using ambient light from both daylight and fluorescent lighting. One of the limitations of this study was that they only compared this multi-site PTT result with an ECG–PPG-derived PTT. They did not compare the PTT with any measurements of BP.

One of the earliest published multi-site PPG studies, McCombie et al. [37], demonstrated how multiple PPG probes can be attached to a single artery on the hand in the form of an extended glove. Although the PTT delay is quite short, the current microcontroller technology can readily measure pulse timings with a microsecond accuracy and thus can be used to estimate BP changes.

Similarly, Nabeel et al. [16,29,34,36], explored ways to collect multi-site PPG using only one probe on one part of the body. They used a single device with one infrared (IR) LED and two PPG probes that were a fixed distance apart to measure the PTT along a single artery. This solves several issues. First, it eliminates the inaccuracies from estimating the distance between the two PPG probes. Second, when two probes are located on different body parts, there is an inaccuracy associated with the multiple branch points between points A and B and the multiple reflections of the wave. Lastly, only one device is needed. Three of the four studies used a sphygmomanometer as the gold standard, while one used tonometry [29]. Of the four studies, only one had a sample size of 35 normotensive subjects [16]. 

Another two studies, Liu et al. [26,35], explored the use of a single PPG device with multi-wavelength probes for measuring PTT. Signals with shorter wavelengths were dispersed superficially and represented capillaries, while longer wavelengths penetrated deeper and represented arterioles. The time difference between IR-PPG (“arterioles”) and blue-PPG (“capillaries”) was used to calculate PTT. This PTT was then compared with the ECG–PPG PTT. The BP was simultaneously compared with a reference BP. The benefit of this method is that it allows for a small device that can be worn in a single location; one of these studies suggested implementation into a smart watch, ring, or earbud [26]. In addition, this study had four subjects that were undergoing a coronary angiography. A limitation of these studies was that they used the finger clamp method as the gold standard.

### 4.3. Imaging PPG

More recently, it has been demonstrated that the optical pulse perfusion in the human skin can be measured with a camera [38]. This technique is now known as “imaging PPG” (iPPG) or “video plethysmography.” By processing the separate color channels in the camera image, the separate color channels can be combined using blind source separation methods to reconstruct the pulse waveform. Since a camera can measure the pulse at multiple points on the body simultaneously, researchers have developed a variety of methods using iPPG to estimate blood pressure. 

Hybrid techniques have also been developed. Zhang et al. [33] proposed a possible smart watch application using contact PPG and iPPG. The smart watch would contain a contact PPG probe for the wrist and a camera to analyze the face for an iPPG when the watch is raised towards head level. The iPPG would then use computer software to analyze the changes in light on the skin surface to detect the pulse wave. However, the iPPG was collected under a single fluorescent lamp only, and the contact PPG used in the experiment was a normal finger PPG. It is unclear how the system would perform under the varying spectrum of possible lighting scenarios in day-to-day living. Still, this study provides a proof of concept for a nonintrusive method of obtaining two PPG signals to calculate BP. The iPPG was first demonstrated on a smart phone by Poh et al. [39]. Fletcher et al. was one of the first to investigate the effect of different ambient lighting on iPPG using an Android phone [40]. A recent iPPG study [41], with the use of a smartphone camera, showed promising results toward measuring blood pressure; however, the data were collected only from normotensive subjects. 

### 4.4. Mitigating Motion Artifacts

Only one study, Wang et al. [25], addressed the issue of motion artifacts, a key challenge to reliable clinical measurements with the PPG. Their study used two PPG sensors (one on the arm, one on the wrist) and an accelerometer at the wrist. The accelerometer was used to identify and remove motion artifacts from the PPG signal. The study was able to show reliable BP measurements while subjects were walking with a normal arm swing. While this study was performed under controlled laboratory settings that did not fully encompass the range of movement in day-to-day living, it did show that motion artifacts can often be accounted for and its effects mitigated via signal processing, but that this is a key area of development needed for ambulatory PPG-based BP assessments.

## 5. Recommendations

As mentioned in the introduction, a vast majority of the publications use the term “multi-site PPG” to describe a dual PPG system. In our literature search, there was only one study that used more than two PPGs in the analysis of BP. This study used PPG analysis of three facial regions in combination with machine learning in order to develop an algorithm for BP prediction [24]. In addition, PTT and PAT are routinely used interchangeably. Our recommendation is:

(1) to apply all recommendations in Elgendi [12] but in multiple PPG configurations,

(2) to compare the performance features extracted from signal PPG signals and multiple PPG signals for assessing BP,

(3) to optimize the locations of measurement for extracting PTT that are correlated with BP,

(4) to include more unhealthy subjects with co-morbidities,

(5) to include subjects from different age ranges,

(6) to explore different noisy environments, and

(7) to investigate the integration of multiple PPG sensors (contact and non-contact) in intelligent clothing or wearable devices.

A thorough literature search using PubMed revealed only 13 publications from January 2010 to January 2019 regarding the use of multi-site PPG for BP estimation. The research suggests that PPG simultaneously from a range of body sites is a promising approach for the continuous tracking of BP. The current research landscape is lacking given the increasing need for a technology to continuously and non-invasively monitor BP. The main shortcoming is that a majority of research studies do not have large enough sample sizes, nor do they include participants with co-morbidities. That being said, a surprising number of studies used a method of BP provocation, showing that their approaches are sensitive to changes in BP. There were also many different approaches to creating a device that would be convenient to wear on a daily basis. The direction of future research should be toward validating the proposed technologies using larger sample sizes and broader populations.

## 6. Conclusions

A systematic review of multi-site PPG-based blood pressure measurement studies from January 2010 to January 2019 was conducted, revealing that there were many studies with promising PPG technologies, but were not mature enough to make a powerful and statistically significant contribution toward the reliable non-invasive measurement of arterial blood pressure. This review creates the necessary motivation for research groups around the globe to intensify their efforts toward the creation of a much needed disruptive technology offering non-invasive, cuff-less, continuous, and calibration-free measurement of blood pressure.

## Figures and Tables

**Table 1:** Summary of findings of all papers included in this review.

Year	Authors	^#^ Subjects	Age Range of Subjects (Years)	Gold Standard	Parameters Measured	Mean BP Absolute Difference	Absolute of Value of Correlation Coefficient between Estimated BP (or PTT Feature) and Referenced BP
2018	Liu et al. [26]	*N*_1_ = 10 *N*_2_ = 10	N/R	Finometer	PTT (ECG, ICG, Finger_PPG_) PTT (MW Finger_PPG_)	1.86 mmHg MAP	N/R
		*N*_3_ = 4	N/R	IBP		2.72 mmHg MAP	N/R
2018	Wang et al. [25]	*N*_1_ = 30	20–27	BP Cuff	PTT (Wrist_PPG_ to Arm_PPG_ with MA)	N/R	*r* = 0.75 SBP *r* = 0.78 DBP
2018	Viejo et al. [24]	*N*_1_ = 15	20–38	BP Cuff	PTT (2 cheeks_PPG_, forehead_PPG_)	N/R	*r* = 0.85 BP
2017	Nabeel et al. [16]	*N*_1_ = 35	23–32	BP Cuff	PTT (Carotid_PPG_ to Carotid_PPG_) PTT (Carotid_PPG_ to Finger_PPG_) PTT (ECG to Carotid_PPG_)	N/R	*r* = 0.74 SBP *r* = 0.77 DBP *r* = 0.78 MAP
2017	Nabeel et al. [29]	*N*_1_ = 5	24–30	Tonometry	PTT (Carotid_PPG_ to Carotid_PPG_)	N/R	N/R
2017	Beckmann et al. [30]	*N*_1_ = 5	25–36	N/R	PTT (Wrist_PPG_ to Index Finger_PPG_) PTT (Wrist_PPG_ to Middle Finger_PPG_) PTT (Wrist_PPG_ to Ring Finger_PPG_) PTT (Wrist_PPG_ to Little Finger_PPG_)	N/R	N/R
2017	Zhang et al. [33]	*N*_1_ = 29	20–30	BP Cuff	PTT (Face_PPG_ to Finger_PPG_) PTT (Temple_PPG_ to Wrist_PPG_)	N/R	*r* > 0.6 SBP in 75.9% subjects
2016	Nabeel et al. [34]	*N*_1_ = 17	21–34	BP Cuff	PTT (Carotid_PPG_ to Carotid_PPG_)	N/R	*r* = 0.68 SBP *r* = 0.71 DBP *r* = 0.72 MAP
2016	Liu et al. [35]	*N*_1_ = 10	22–26	Finometer	PTT (ECG to Finger_PPG_) PTT (MW Finger_PPG_)	N/R	*r* = 0.76 SBP
2015	Nabeel et al. [36]	*N*_1_ = 13	22–31	BP Cuff	PTT (Carotid_PPG_ to Carotid_PPG_)	N/R	*r* = 0.5 SBP *r* = 0.66 SBP *r* = 0.63 MAP
2015	Liu et al. [31]	*N*_1_ = 12	24–35	ECG–PPG PAT	PTT (Temple_PPG_ to Index Finger_PPG_)	N/R	N/R
2011	Chen et al. [18]	*N*_4_ = 35	17–21 and 58–62	IBP	PTT (Ear_PPG_ to Toe_PPG_)	2.16 mmHg SBP 1.49 mmHg DBP	N/R
2010	Proenca et al. [32]	*N*_1_ = 20	20–37	Finometer	PTT (ECG, ICG, unspecified PPG) PTT (Ear_PPG_ to Finger_PPG_)	N/R	*r* = 0.22 SBP

*N*_1_ = normotensive subjects, *N*_2_ = hypertensive subjects, *N*_3_ = patients undergoing coronary angiography, *N*_4_ = patients under general anesthesia, *r* = Pearson’s correlation coefficient, Finometer = finger blood pressure, IBP = invasive BP, MW = multi-wavelength, MA = motion artifact, ECG = electrocardiogram, ICG = impedance cardiography, PTT = pulse transit time, N/R = not reported, SBP = systolic BP, DBP = diastolic BP, MAP = mean arterial pressure, Finger_PPG_ = PPG collected from a finger.

## Appendix

The titles (translated into English) of articles that are not accessible in English language:

(Clinical effect of lower tidal volume combined with lung recruitment maneuver on ARDS for post-operative esophageal carcinoma surgery patients).

(Comparison of early retinal microvascular changes and microalbuminuria as indicators for increased cardiovascular risk).

(Pilot study: Wrist digital sphygmomanometers as an alternative for non-invasive blood pressure measurement in pediatric population).

(Ventilation strategies for chronic obstructive pulmonary disease).

(Results of using the dosing Valsalva–Weber test to determine autonomic disorders in patients with vasovagal syncope).

(Monitoring oxygen consumption in energy metabolism in pediatric anesthesia: clinical utility).

(Pulmonary hypertension: a rare cause of unexplained dyspnea).

(Minimally invasive treatment of Dupuytren’s contracture using Pi needle percutaneous multi-segmental fasciotomy).

(Analysis of thyroid homeostasis disorders in patients with severe traumatic brain injury).

(Central sleep apnea (Ondine’s curse syndrome) in medullary infarction).

(The coronary patient six months after cardiac rehabilitation: rehabilitation evaluation research (RER study)).
